# Automatic classification of cells in microscopic fecal images using convolutional neural networks

**DOI:** 10.1042/BSR20182100

**Published:** 2019-04-05

**Authors:** Xiaohui Du, Lin Liu, Xiangzhou Wang, Guangming Ni, Jing Zhang, Ruqian hao, Juanxiu Liu, Yong Liu

**Affiliations:** School of Optoelectronic Information, MOEMIL Laboratory, University of Electronic Science and Technology of China, Chengdu 610054, China

**Keywords:** cell object detection, deep learning, fecal microscopic images, image recognition, pattern recognition

## Abstract

The analysis of fecal-type components for clinical diagnosis is important. The main examination involves the counting of red blood cells (RBCs), white blood cells (WBCs), and molds under the microscopic. With the development of machine vision, some vision-based detection schemes have been proposed. However, these methods have a single target for detection, with low detection efficiency and low accuracy. We proposed an algorithm to identify the visible image of fecal composition based on intelligent deep learning. The algorithm mainly includes region proposal and candidate recognition. In the process of segmentation, we proposed a morphology extraction algorithm in a complex background. As for the candidate recognition, we proposed a new convolutional neural network (CNN) architecture based on Inception-v3 and principal component analysis (PCA). This method achieves high-average *Precision* of 90.7%, which is better than the other mainstream CNN models. Finally, the images within the rectangle marks were obtained. The total time for detection of an image was roughly 1200 ms. The algorithm proposed in the present paper can be integrated into an automatic fecal detection system.

## Introduction

Microscopic examination is an important method of clinical testing. Medical staff can determine a patient’s pathological changes based on the fecal routine, by counting the number and the type of cells under a microscope to understand and help analyze and diagnose disease. The majority of small hospitals conduct biological cell detection by manual method. This kind of detection method obviously has the problem of insufficient speed and precision. The rapid identification of the visible components of microscopic cell images in medicine has been the key to the detection of microscopic cells. With the development of machine vision research and improvement in biomedical image processing technology, medical microscopic image processing technology has gradually developed from the traditional, manual recognition method to automated computer identification. With machine vision at the core, image processing technology has become the focus of current research on the automatic identification of visible components of microscopic cell images.

Habibzadeh et al. [1] describe a subcomponent system for the automatic classification of a complete blood count. They compared three different methods: support vector machine (SVM) using standard intensity and histogram features (IHF); SVM with features extracted by a kernel principal component analysis (PCA) of the IHF; and convolutional neuron network (CNN), and determined that CNN was the best one. The CNN method is not conclusively the best as the number of samples is small (115 training and 25 testing). Gautam et al. [2] proposed a method to detect leukocytes in human blood. They simply segmented an image by Otsu thresholding and selected the composition that looked like leukocytes by morphing, finally classifying the sample by naive Bayes algorithm. However, their dataset was poor, with an accuracy of 80.88%. Liu et al. [3] proposed an artificial neural network (ANN) to classify fungi with ten morphological features, which achieved an accuracy of 94.5%. But features extracted by morphology have a certain degree of subjectivity and lack of representation. Rosyadi et al. [4] used five kinds of features: normalized area, circularity, eccentricity, normalized parameter, and solidity, and varied their types and their degree of influence. Then they use k-means clustering to classify the cell, with an accuracy of 67%. Manik et al. [5] extracted the cell from a colorful image using the segment and morphology method, then computed eight different features for the ANN, and finally got an accuracy of 98.9% from a total sample of 90. Zhang et al. [6] combined two methods to distinguish the leukocytes and impurities. One method was feature extraction and SVM, which reached an accuracy of 92.5%; the other method was CNN, with an accuracy of 89.5%. They combined the two approaches and achieved an accuracy of 93.5%.

Regarding the detection of the objects in microscopic images, the microscopic image components of stool are more complex. In general, these images contain a lot of impurities. Due to the different shapes of these impurities, some impurities are similar to the cells sought for detection, which makes it difficult to detect the real samples. It is difficult to achieve high accuracy using the traditional morphological detection method.

In recent years, researchers have been applying deep learning methods, thus creating a breakthrough in the field of artificial intelligence detection toward biological image intelligence detection, with remarkable results. Ishikawa et al. [7] presented a novel method to robustly segment cell regions using binarized normed gradients (BING) objectness estimation and CNN. Experimental results showed an accuracy of 98.5%. Zhang et al. [8] proposed a deep detector for cells based on the framework of Faster R-CNN, and on this basis presented a Circle Scanning Algorithm (CSA) for the redetection of adhesion cells. Albayrak et al. [9] extracted the features by CNN, and a combination of PCA and linear discriminant analysis (LDA) dimension reduction, then used the SVM for final classification of mitotic and non-mitotic cells.

Methods of deep learning such as Faster R-CNN [22], YOLO [13], SSD [12] have high detection and recognition accuracy; however, they are very dependent on the number of sample sets. These models are easy to overfit when the target sample size is not large enough. The more complex and expressive the model, the easier it is to sacrifice the interpretation ability of future data and focus on interpretation training data. Deep learning is often used in higher dimensional learning, but the number of samples required increases exponentially with an increase in dimensions. While many researchers have proposed solutions to overfitting, namely data augmentation [10–15], the problem of overfitting still cannot be solved while the sample size is small.

In order to detect the number and position of the constituent elements in a microscopic image (including erythrocytes, leukocytes, and molds), we separated the detection into two parts. One is the candidate segmentation, which is the region of cells without labels. The other one is the recognition of the candidates. The algorithm has the higher average *Precision* [with intersection over Union (IOU) > 0.7], which can detect and locate red blood cells (RBCs), white blood cells (WBCs) and molds rapidly. The average *Precision* is around 90.7%, and the detection time is 1200 ms for an image (1600*1200 resolution).

This article is organized as follows. Materials and methods are described in section 2. Section 3 introduces the components of the developed cell detection method, including candidate segmentation and candidate recognition. The setup and results of the experiments and discussions are described in detail in section 4. Conclusions are provided in section 5.

## Materials and methods

**Figure 1:**
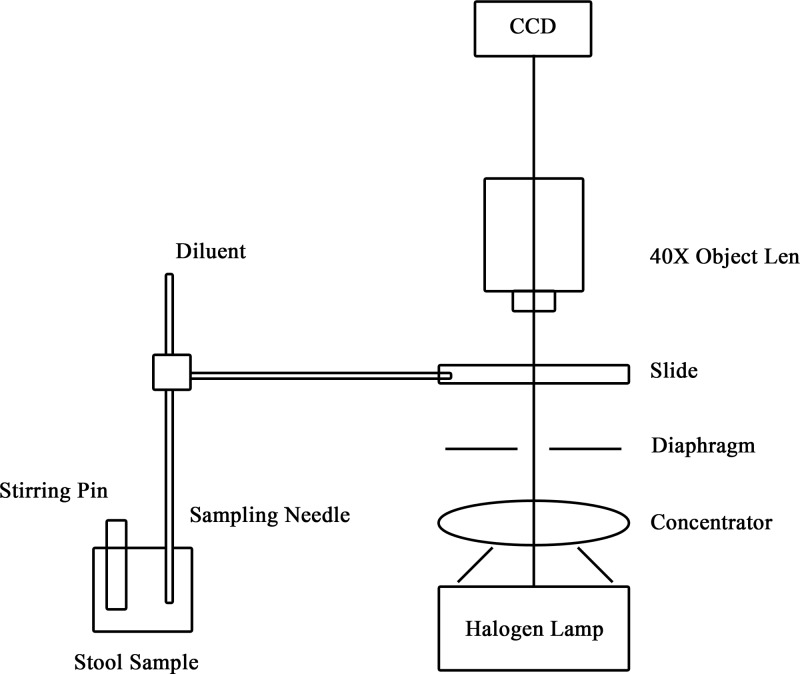
The sample pre-processing and capturing optical system

In our study, we collected 17933 samples from the Sixth People’s Hospital of Chengdu, Sichuan Province. The collected stool samples were stirred well by the equipment, filtered with a strainer, set aside, poured into a flow cell, and the images were collected by a microscope (totaling 89665 images). The design of sample pre-processing and capturing optical system is shown in Figure 1.

The capture environment was a biological microscope and a charge-coupled device (CCD) camera, which were used to obtain microscopic cell images. We used an OLYMPUS CX31 biological microscope with a 40× objective lens (numerical aperture (NA): 0.65, material distance: 0.6 mm). An EXCCD01400KMA CCD camera with a pixel size of 6.45 × 6.45 µm was used for exposure. After the cell boxing by clinical doctors, we obtained 4459 RBCs, 4305 WBCs, and 6536 molds as ground-truth.

### Dataset split

We use a 4:1:1 split for training set, validation set and testing set. Cross-validation was used when evaluating performance.

### Negative selection

As for the negative candidates, we tried to select noise that was similar to the positive candidates, and added some other ingredients. The strategy for the impurity selection was as follows:

- Random impurity candidates;
- Candidates that were similar to positive candidates. For example, spores are similar to RBCs to some extent, and concentrated cells are similar to WBCs.

**Figure 2:**
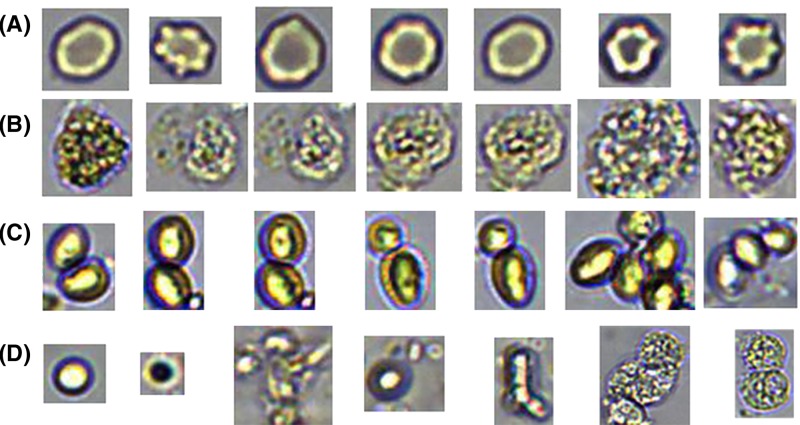
Cell candidates extracted (**A**) Red blood cells; (**B**) White blood cells; (**C**) Mildews; (**D**) Impurities.

The typical extracted candidates set is shown in Figure 2:

### Data augmentation

In order to make the classification model more robust to various shapes and forms, and avoid overfitting, each candidate cropped from the original image was pre-processed by the following options:

- Used the original cropped image (marked as *f*);
- Randomly sampled a patch from *f*, where the minimum Jaccard overlap to the *f* is 0.8. Each sampled patch was resized to a fixed size and was horizontally or vertically flipped with a probability of 0.5.

#### Cell location and classification

The cell detection algorithm consisted of two modules: The first was a region proposal, which generated the category-independent candidates. The second was the feature extractor, with a deep convolutional neural network for each candidate and classification.

##### Region proposal

**Figure 3:**
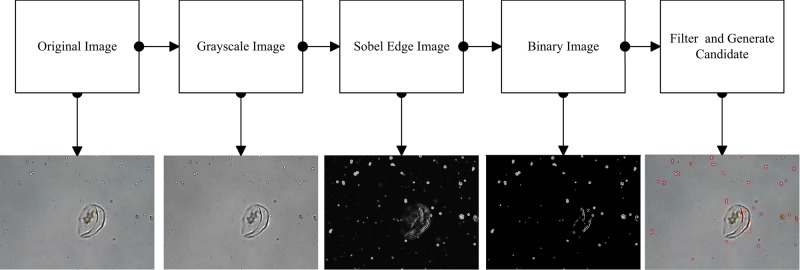
Flow chart of the object segment algorithm

As the composition of the fecal samples was characteristically small in volume, common ratio imaging could not produce the ingredients in a refined pattern. Medical microscopic images of fecal samples have complex backgrounds, forms, blurred image edges and characteristically complex boundary topology. Thus, it is necessary to perform pre-processing when extracting the region proposal. We processed the images thusly:

- The original image was in 24-bit color.
- Sobel operators were applied to filter the image. We used four different operators with four different orientations, as follows:
  $$\begin{array}{llllllllllll} 0 & 1 & 2 & -1 & 0 & 1 & 1 & 2 & 1 & -2 & -1 & 0\\ -1 & 0 & 1 & -1 & 0 & 2 & 0 & 0 & 0 & -1 & 0 & 1\\ -2 & -1 & 0 & -1 & 0 & 1 & -1 & -2 & -1 & 0 & 1 & 2 \end{array}$$
  By combining the four different operator images with the method of maximum, we obtained the marginalized image in Figure 3.
- The binary marginalized image with a local mean threshold was formulated as follows:
  (1) $$\begin{array}{l} th(x,y)=\frac{1}{R^{2}}\sum_{m=-R/2}^{R/2}\sum_{n=-R/2}^{R/2}f(x+m,y+n)\\ g(x,y)=f(x,y)>th(x,y)*rato?255:0. \end{array}$$
  where *th*(x, y) is the local threshold for the input *f*(*x, y*).
- We labeled the connecting component areas after closing the binary image with the structural element 3*3. Regions were filtered if their area and bounding box did not match the condition:
  (2) $$\begin{array}{l} 100<area<8000\\ 30<width(or\;height)<200. \end{array}$$
  All these parameters were statistically obtained from all these datasets.
- We merged the connection regions’ location information from step 4 and step 5. Duplicate location information was then removed.

The algorithmic process and intermediate results during processing each steps of whole proposed algorithm are shown in Figure 3.

##### Object recognition

**Figure 4:**
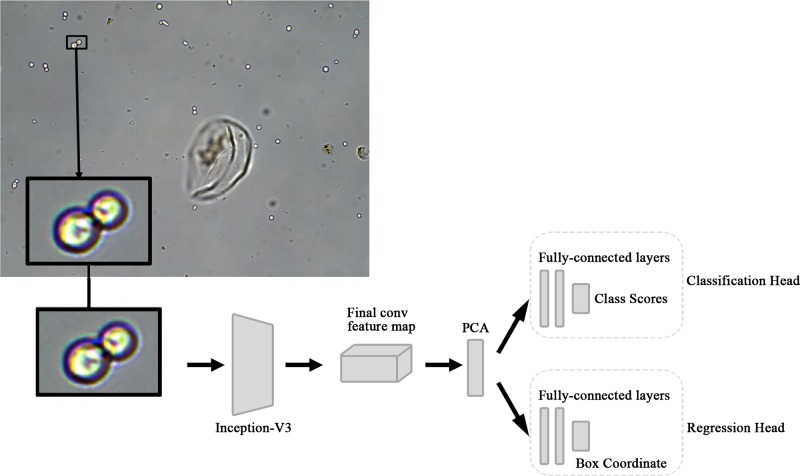
Cell detection structure with CNN model

The recognition of the cell candidates was conducted by CNN models. In the present paper, we designed a new CNN recognition model, based on the inception [21] structure. The model design pipeline is shown in Figure 4.

The segmented candidate regions are sent into the Inception-v3 network for feature extraction. In the last feature map layer, PCA is used to reduce the dimension of the feature vectors. After dimension reduction, the feature vectors are sent to the classification network and the regression network, respectively. The classification network is used for type recognition, while the regression network is used for location correction.

The input dimension of Inception-v3 was [299, 299, 3]. However, the size of the extracted cell candidate sets was different; for example, the size of red blood cells was about [48, 48, 3], the white blood cells were about [58, 58, 3], and the size of the mold varied from [32, 32, 3] to [108, 108, 3]. Thus, the cells that were sent to the CNN network needed to be shrunk. Bilinear interpolation method was used to enlarge the candidate region’s short edge to 299, while the long side was scaled to keep the aspect ratio equal. That is,

(3) [W,H]→[W*H/299,299],if W≥H

where the size of the network is [*W, H*] (*W*>*H*), *H* is retraced to 299, and *W* is equal-ratio scaled.

**Figure 5:**
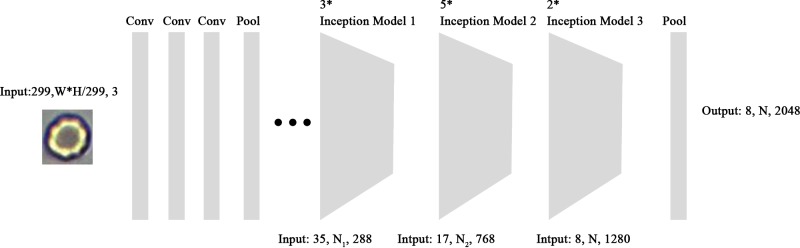
Structure of Inception-v3

The mainstream of CNN was based on Inception-v3. We regarded the last pooling layer of the Inception-v3 network as the feature extraction layer. The structure of Inception is shown in Figure 5.

The size of the feature map is: [8, N, 2048].

Region of interest (ROI) pooling strategy in Fast R-CNN [11] is a method to unify feature dimensions. Although the calculation of ROI Pooling is small, important features are lost to some extent. In the present paper, PCA strategy was adopted in order to retain features to a great extent. Experiments show that this method can effectively improve recognition rate. To reduce feature dimension, it was necessary to stretch the [8, N, 2048] dimension vectors to one dimension [8*N*2048]. The length of the feature vector after stretching was different due to the different size of the input, and thus the dimension of the feature map was different. The principle of PCA processing is to extract the first 1024 eigenvectors. The detailed steps are as follows:

- Create the covariance matrix *S* of eigenvector *X*[(8*N*2048)]:
  (4) $$S={\left(X-\bar{X}\right)}^{T}(X-\bar{X})$$
  where $\bar{X}$ is the mean of the feature.
- Calculate eigenvalues *λ_i_* and eigenvectors *e_i_* for *S*;
- Eigenvalues *λ_i_* are sorted in descending order;
- Select the first 1024 eigenvalues *λ_i_* and eigenvectors *e_i_*;
  (5) $$U=\{e_{1},e_{2},\cdots ,e_{1024}\}$$
  where *U* is the matrix composed of *e_i_*.
- The output can be described as:
  (6) $$X^{\prime }=U^{T}X$$

Experiments show that the first 1024 eigenvectors can retain the characteristic information to a maximum of 97% for all samples. This method not only achieves the purpose of unifying the feature dimension, but also applies all the information in the feature map to a great extent.

##### Training

Due to the introduction of PCA, the entirety of the network training cannot achieve end-to-end training; therefore, we split the training into two modules.

Module 1 was a traditional Inception-v3 network, and the initial parameters of the network were trained by ImageNet dataset [18], that is, transfer learning. As the size of the extracted samples was inconsistent, it was reduced to [299, 299, 3] by bilinear interpolation. By fine-tuning the network, we obtained a module used for classifying the different type of cells, with the input size [299, 299, 3].

**Figure 6:**
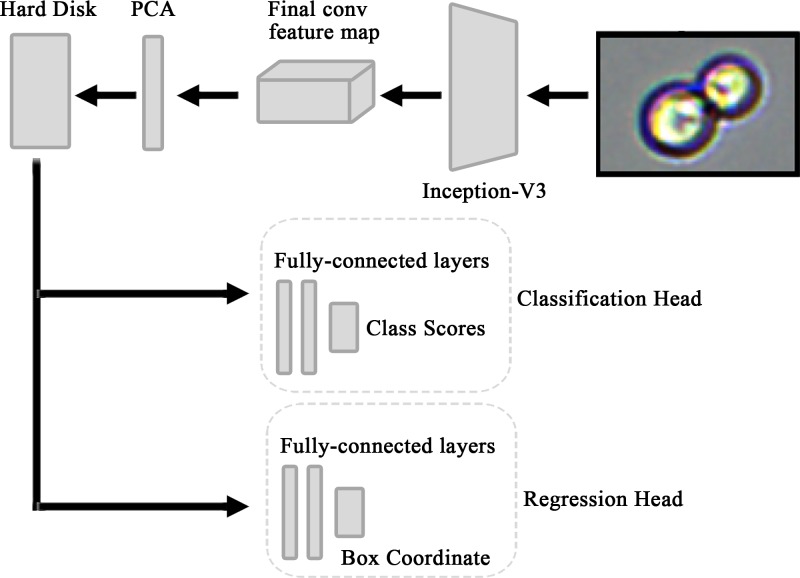
PCA-inception training model

The training of module 2, as shown in Figure 6, sent different sizes of samples to the network trained in module 1 to extract feature information for the feature-map layer. The PCA algorithm was used to compress the feature information into 1024 dimensions, which was saved to a hard disk. According to the preserved feature data, the final model was obtained by training classifier and regressor, respectively.

With the candidate from section 2, it was easy to end-to-end train the CNN architecture in module 1. The loss function is defined as:

(7) $$L(s,t_{x,y,w,h})=L_{cls}(s_{c^{*}})+\lambda [c^{*}>0]L_{reg}(t,t^{*})$$

where c* is the candidate’s ground-truth label, *L_cls_*= −log(s_c*_) is the classification loss (cross-entropy loss), and *L_reg_* is the box regression loss. [c* > 0] is the positive candidates sample. λ is the coefficient of regression loss, which controls the balance of two losses. The *L_reg_* is smooth *L1* loss [11]. There is no regulation loss in loss function and the momentum of 0.9 is used.

##### Evaluation

The classification and positioning of the model in the target detection problem needs to be evaluated, and each image may have different targets for different categories. An IOU between 0 and 1 is the ratio between the intersection and union of the detection boxes predicted by the model and the ground truth, which is also known as the Jaccard index. The higher the IOU, the more accurate is the position of the prediction box. And the IOU can be described as:

(8) $$IOU=\frac{Area(R_{p}\cap T_{gt})}{Area(R_{p}\cup T_{gt})}$$

where $R_{p}\cap \;R_{gt}$ represents for the intersection of the predicted box and ground-truth box. And $R_{p}\cup \;R_{gt}$ represents for the union.

*Precision* and *Recall* are the common metrics for object detection, which can be written as:

(9) $$Precision=\frac{TP}{TP+FP}$$

(10) $$Recall=\frac{TP}{TP+FN}$$

where *TP* (true positive) indicates the box is correctly predicted as the ground-truth (IOU > 0.7). *FP* (false positives) refers to the background predicted as the objects. *FN* (false negative) is the ground-truth object detected as the background. And there were no *TN* in object detection. The *F_1_* score is the harmonic mean of the *Precision* and *Recall*; the higher the *F_1_*, the higher the accuracy:

(11) $$F_{1}=\frac{2*Precision*Recall}{Precision+Recall}$$

## Results and discussion

### Region proposal

For the extraction of region proposal, selective Search (SS) is used for comparison to analyze the advantages of this algorithm in generating candidates in fecal microscopic images. SS is widely used in the object recognition field. It was first proposed by J.R.R. Uijlings in 2012 [16] and has been well applied in the both R-CNN [17] and Fast R-CNN [11]. The SS method is more suitable for segmentation and extraction of large targets on small images such as the virtual object classes (VOC) or the ImageNet Large Scale Visual Recognition Challenge (ILSVRC), while the extraction of tangible components such as cells does not apply, as shown in Tables 1 and 2.

**Table 1:** Segment result of five different samples

ID	Algorithm	Number of targets of ground truth	Number of candidates	Number of missing	Time consumed (ms)
1	2A	6	19	0	534.746
	2B		22	0	3858.38
2	2A	5	16	0	986.883
	2B		16	2	3462.25
3	2A	10	91	0	838.002
	2B		105	0	4131.89
4	2A	13	93	0	627.661
	2B		98	2	3832.61
5	2A	11	119	0	982.831
	2B		106	1	3797.69

2A: the result for the object segment algorithm; 2B: the result for the SS.

**Table 2:** Segment result statistics of 89665 different images

Algorithm	Total target by artificial	Total missing	Average number of candidates per image	Average time consumed (ms)
2A	15818	210	65.41	648.808
2B		739	70.35	3916.31

2A: the result for the object segment algorithm; 2B: the result for the SS.

**Figure 7:**
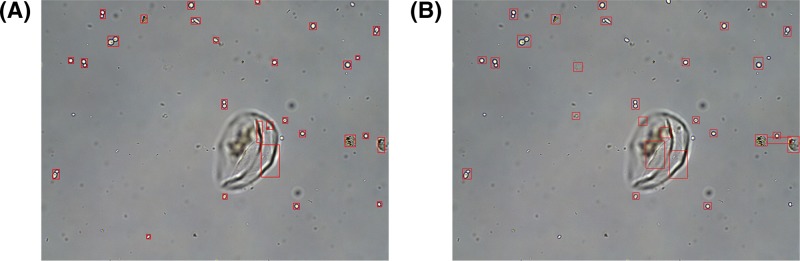
The comparison of the proposed method and SS: (**A**) proposed method; (**B**) SS

The method proposed has a lower missing detection rate of 1.3%, and the efficiency is six times greater compared with the SS method. The results of the two methods are shown in Figure 7.

As compared with the same morphological method used in context [6], the segmentation method we used only had four operators in different directions, with faster calculation speeds and more accurate segmentation. We also tested the bottom-hat transform method [3] used for segmentation. This method is very good for the detection of fungi, but there are many missed samples for other structured components, as the edge of mildews is generally relatively bright or dark, which is not easy to detect due to adhesion with surrounding impurities. With respect to multi-target detection, it does not suitable for segmentation.

### Object recognition

**Figure 8:**
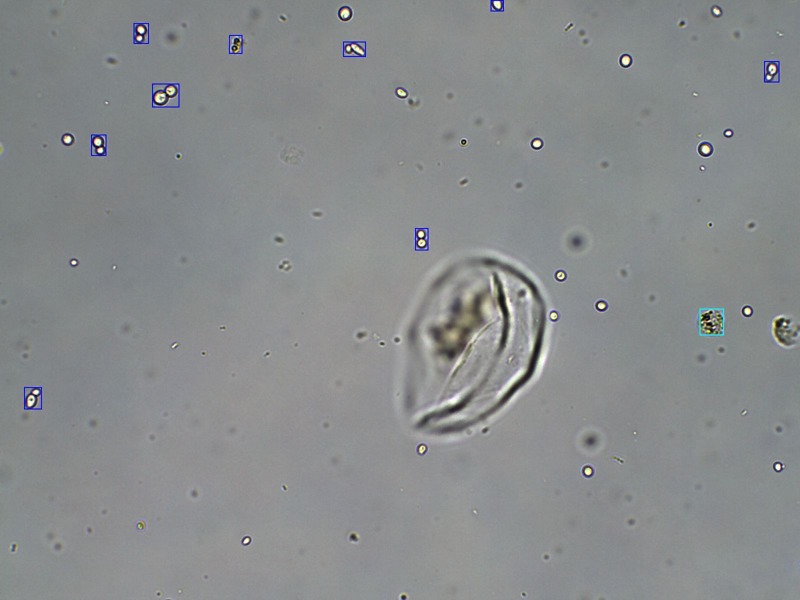
Image recognition result: the blue boxes represent molds, while the cyan box is a WBC

A whole image recognition effect is shown in Figure 8; the testing images can be any size.

For the testing set, the results of detection are in Table 3.

**Table 3:** The recognition results of PCA-Inception model

	RBCs	WBCs	Molds	Total
Annotation Number	761	693	1055	2509
True positive	728	611	983	2322
*Precision*	92.9%	88.8%	90.3%	Ave: 90.7%
*Recall*	95.7%	88.2%	93.2%	Ave: 92.5%
*F_1_*	94.3%	88.5%	91.7%	Ave: 91.6%

In terms of target recognition, we proposed a PCA–CNN strategy based on the latest Inception network architecture, which is superior to the traditional network. Compared with the method used by Habibzadeh et al. [1], which requires very reasonable features in combination with SVM to obtain classification results, and the method of man-made extraction of morphological characteristics proposed in the literature [3–5], the algorithm-acquired features we proposed are more representative of migration learning extracted in the last feature map of CNN. This is because Inception simulates the human local perception to the target with the use of convolution, and Inception uses 1 * 3 and 3 * 1 convolution kernels, which can significantly reduce the training parameters, improve training and recognition efficiency. At the same time, Inception has the wider and deeper network architecture. The CNN model is used in the literature [6], but the CNN structure used is too simple and has many parameters. It is efficient for single-target detection but it is not suitable for multi-target detection.

At the same time, we used the candidate sets extracted by morphological methods to test with VGG-19 [19], Inception-v3 [20], Inception-v4 [21], and Inception-Resnet-v2 [22]. The results are shown in Table 4.

**Table 4:** Comparison of the recognition results of several models

	VGG-19	Inception-v3	Inception-v4	Inception-Resnet-v2	PCA-Inception-v3
Average *Precision*	83.7%	89.6%	89.2%	89.8%	90.7%
Average *Recall*	86.2%	90.1%	90.8%	90.4%	92.5%
Average *F_1_*	84.9%	89.8%	90.0%	90.1%	91.6%

The comparison shows that the model we proposed has improved in terms of *Precision* and *Recall*.

## Conclusion

In summary, we presented a model of a cell object detection method in fecal microscopic images. This method used morphological methods to extract the candidates in a complex background, and then used the PCA-Inception-v3 architecture for recognition and location. The method can be applied to different-size images, with a high average *Precision* of 90.7% and low-time consumption (1200 ms). The biomechanical component detection algorithm described in this paper has been applied to micro-imaging intelligent devices, and achieved good clinical test results.

## Abbreviations

ANN: artificial neural network
CNN: convolutional neural network
IHF: intensity and histogram feature
IOU: intersection over union
PCA: principal component analysis
RBC: red blood cell
R-CNN: regions with convolutional neural network
ROI: region of interest
SS: selective Search
SSD: single shot multibox detector
SVM: support vector machine
WBC: white blood cell
YOLO: you only look once: unified, real-time object detection
